# Development of a Pan-H1 Influenza Vaccine

**DOI:** 10.1128/JVI.01349-18

**Published:** 2018-10-29

**Authors:** Nicole Darricarrère, Svetlana Pougatcheva, Xiaochu Duan, Rebecca S. Rudicell, Te-Hui Chou, Joshua DiNapoli, Ted M. Ross, Tim Alefantis, Thorsten U. Vogel, Harry Kleanthous, Chih-Jen Wei, Gary J. Nabel

**Affiliations:** a Sanofi, Cambridge, Massachusetts, USA; b Sanofi Pasteur Biologics, Cambridge, Massachusetts, USA; c Center for Vaccines and Immunology, Department of Infectious Diseases, University of Georgia, Athens, Georgia, USA; St. Jude Children's Research Hospital

**Keywords:** H1N1 influenza, HA-ferritin nanoparticles, vaccines, H1N1, influenza vaccines

## Abstract

The efficacy of current seasonal influenza vaccines varies greatly, depending on the match to circulating viruses. Although most vaccines elicit strain-specific responses, some present cross-reactive epitopes that elicit antibodies against diverse viruses and remain unchanged and effective for several years. To determine whether combinations of specific H1 hemagglutinin (HA) antigens stimulate immune responses that protect against diverse H1 influenza viruses, we evaluated the antibody responses elicited by HA-ferritin nanoparticles derived from six evolutionarily divergent H1 sequences and two computationally optimized broadly reactive antigen (COBRA) HA antigens. Humoral responses were assessed against a panel of 16 representative influenza virus strains from the past 80 years. HAs from the strains A/NewCaledonia/20/1999 (NC99), A/California/04/2009 (CA09), A/HongKong/117/1977 (HK77), COBRA X6, or P1 elicited neutralization against diverse strains, and a combination of three wild-type HA or two COBRA HA nanoparticles conferred significant additional breadth beyond that observed with any individual strain. Therefore, combinations of H1 HAs may constitute a pan-H1 influenza vaccine.

**IMPORTANCE** Seasonal influenza vaccines elicit strain-specific immune responses designed to protect against circulating viruses. Because these vaccines often show limited efficacy, the search for a broadly protective seasonal vaccine remains a priority. Among different influenza virus subtypes, H1N1 has long been circulating in humans and has caused pandemic outbreaks. In order to assess the potential of a multivalent HA combination vaccine to improve the breadth of protection against divergent H1N1 viruses, HA-ferritin nanoparticles were made and evaluated in mice against a panel of historical and contemporary influenza virus strains. Trivalent combinations of H1 nanoparticles improved the breadth of immunity against divergent H1 influenza viruses.

## Abstract (precis)

Seasonal influenza vaccines elicit strain-specific immune responses designed to protect against circulating viruses. Because these vaccines often show limited efficacy, the search for a broadly protective seasonal vaccine remains a priority. Among different influenza virus subtypes, H1N1 has long been circulating in humans and has caused pandemic outbreaks. In order to assess the potential of a multivalent HA combination vaccine to improve the breadth of protection against divergent H1N1 viruses, HA-ferritin nanoparticles were made and evaluated in mice against a panel of historical and contemporary influenza virus strains. Trivalent combinations of H1 nanoparticles improved the breadth of immunity against divergent H1 influenza viruses.

## INTRODUCTION

Despite the availability of a seasonal vaccine, influenza remains a public health burden that disproportionally affects the young and elderly (1, 2). H1N1 influenza has caused pandemic outbreaks, which can arise when new strains appear or herd immunity has waned (3). There remains a pressing need to improve the efficacy of influenza vaccines against influenza A viruses of pandemic potential (4).

Most vaccines today are manufactured by culturing live virus in eggs (5). Egg allergies, egg adaptation mutations, supply shortages, and lengthy production cycles have limited their utility (5–8). Since protection is mainly mediated by neutralizing antibodies against hemagglutinin (HA), recombinant protein vaccines based on purified HA have been developed as alternatives (9, 10). Recently, we demonstrated that HA can be genetically fused to a bacterial ferritin, and this fusion protein self-assembles into a nanoparticle with virus-mimicking structural features that induce protective immunity in mice and ferrets (11). This nanoparticle-based vaccine technology is well suited to optimize immune responses to HA proteins because it allows the generation of novel protein antigens that may not support viral replication.

The discovery of broadly neutralizing antibodies that target the receptor-binding site in the head domain (12–16) or the conserved stem region of HA (17–20) suggests a path toward a universal influenza vaccine. However, these antibodies are underrepresented in humoral responses elicited by the current seasonal influenza vaccines, which often lack breadth of protection beyond the matched strain (9, 10). Some influenza virus strains have remained unchanged in the vaccine formulation for several years, while others are replaced after a single season, raising the possibility that some strains inherently present cross-reactive epitopes more effectively.

Here, we evaluate the immune cross-reactivity of HA sequences from different time periods in an effort to develop a more effective pan-H1 influenza vaccine. We sought to understand the breadth of multivalent combinations of evolutionarily divergent H1 HA antigens that display complementary cross-neutralization profiles. We characterized the breadth of response elicited by eight HA antigens, including six wild-type sequences, representing distinct branches of the evolutionary landscape of H1N1 influenza viruses, and two computationally designed antigens (21), alone or in combination. Combinations of three HA-ferritin nanoparticles (HA-Nps) elicited neutralizing antibody responses against a diverse collection of H1N1 influenza viruses from 80 years of viral evolution.

## RESULTS

### Design, purification, and characterization of HA-ferritin nanoparticles displaying HA proteins from divergent influenza virus strains.

H1N1 strains H1/California/07/2009 (CA09) and H1/New Caledonia/20/1999 (NC99) were selected by the WHO and remained unchanged in the vaccine composition for extended periods, 2010 to 2017 and 2000 to 2007, respectively. The other four HA candidates were selected from a time period prior to the widespread use of influenza vaccines. A/Fort Monmouth/1/1947 (FM47) was selected as a representative of a major antigenic change that resulted in the 1947 pseudopandemic (22). Two more candidates were chosen from a period when H3N2 largely displaced H1N1 influenza: A/Malaysia/302/1954 (MAL54) and A/Denver/1/1957 (DV57). Finally, A/Hong Kong/117/1977 (HK77) represented a candidate from the 1977 H1N1 influenza epidemic (22). In addition to these wild-type sequences, we evaluated two newly described sequences, computationally optimized broadly reactive antigen (COBRA) P1 and COBRA X6, generated through a computational method of hierarchical sequence averaging (21). COBRA X6 was generated from human H1N1 influenza sequences spanning 1999 to 2012, and COBRA P1 was generated from human H1N1 strains spanning 1933 to 1957 and 2009 to 2011 plus swine H1N1 influenza virus strains from 1931 to 1998 (21).

**FIG 1:**
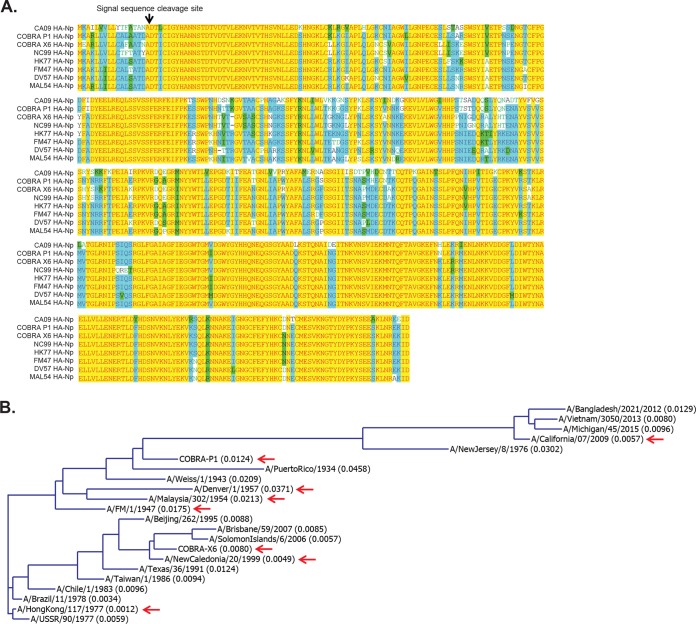
Sequence comparison and homology of representative H1N1 influenza virus strains. (A) Sequence alignment of candidate HA immunogens using vector NTI AlignX software. Red on yellow, consensus residue derived from a completely conserved residue at a given position. Black on green, consensus residue derived from the occurrence of greater than 50% of a single residue at a given position. Green on white, residue weakly similar to consensus residue at given position. Blue on cyan, consensus residue derived from a block of similar residues at a given position. Black on white, nonsimilar residues. (B) A dendrogram generated with the neighbor-joining method (Align X module of Vector NTI software) for the HA protein sequences from the influenza virus strains used in this study. Red arrows indicate candidate antigens. The calculated distances, shown in parentheses following the strain name, are related to the degree of divergence between the sequences in the alignment.

**FIG 2:**
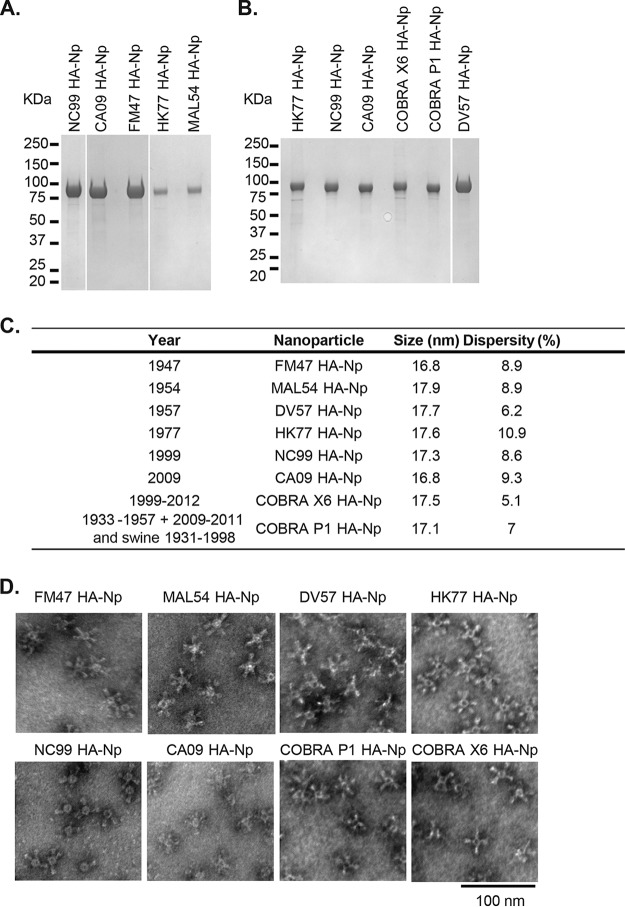
Characterization of self-assembling HA-ferritin nanoparticles derived from diverse influenza virus strains. HA-ferritin nanoparticles presenting six evolutionarily divergent H1 HA antigens and two computationally generated (COBRA) antigens. (A and B) Purity of HA-nanoparticles was assessed by SDS-PAGE Coomassie staining. (C) Nanoparticle size and polydispersity of HA-nanoparticles measured by dynamic light scattering. (D) Nanoparticle integrity was visualized by negative-stain electron microscopy at ×80,000 magnification. FM47, A/Fort Monmouth/1-JY2/1947; MAL54, A/Malaysia/302/1954; DV57, A/Denver/1/1957 (DV57); HK77, A/Hong Kong/117/1977; NC99, A/New Caledonia/20/99; CA09, A/California/4/2009. COBRA P1 and COBRA X6 are computationally generated consensuses from multiple sequences, described recently by Carter et al. (21).

HA-Nps were generated by fusing the HA ectodomain sequences (lacking 48 C-terminal transmembrane residues) (Fig. 1A) to the N terminus of ferritin to produce nanoparticles that self-assembled in mammalian cells and were released into the culture media. The sequence diversity of candidate antigens and assay strains is depicted in a dendrogram generated by sequence alignment with the neighbor-joining method (Fig. 1B). HA-Nps were purified from Expi293 cell culture supernatant by anion exchange and size exclusion chromatography (Fig. 2A and B). The purified HA-Nps displayed the expected size by dynamic light scattering, between 16 and 18 nm (Fig. 2C). The formation of HA-Nps was also confirmed by negative-stain transmission electron microscopy (TEM) (Fig. 2D).

### Immunogenicity of single-strain HA-ferritin nanoparticles against a representative panel of divergent H1N1 influenza viruses.

**FIG 3:**
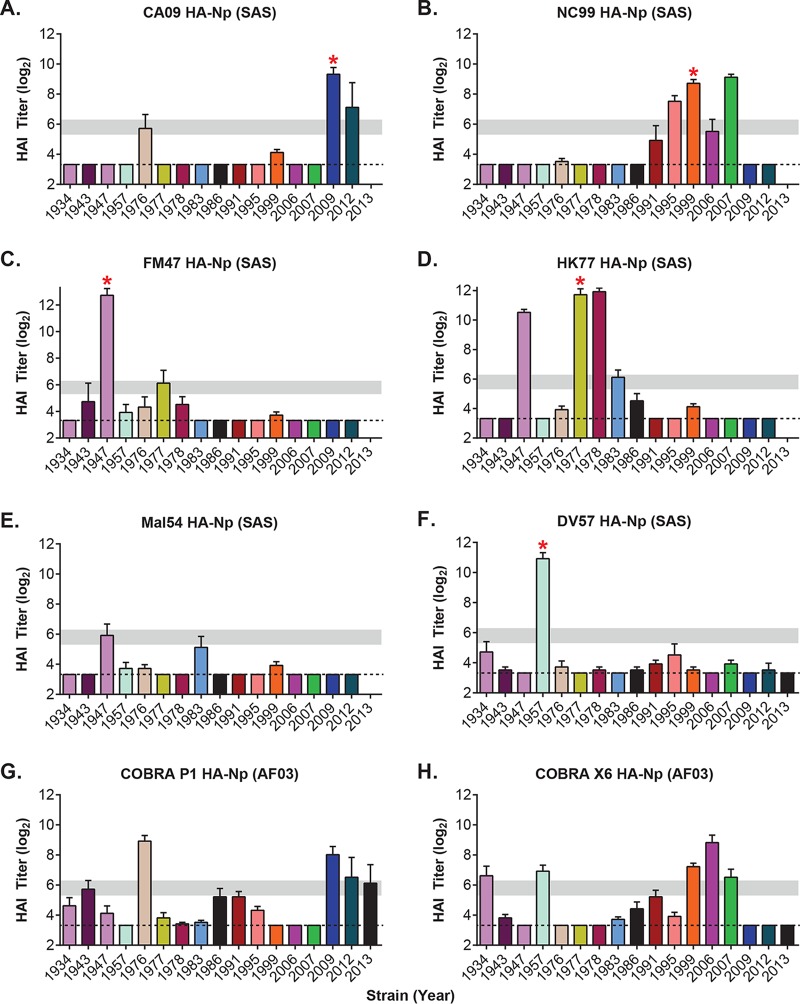
Potency and breadth of immune response elicited by divergent HA-ferritin nanoparticles. Shown are hemagglutination inhibition (HAI) titers (log_2_) of sera from mice 6 weeks after immunization with the indicated HA-Np vaccine against a panel of divergent H1N1 influenza viruses. Mice (*n* = 5) were immunized with HA-Nps at weeks 0 and 3 with either SAS adjuvant (A to F) or AF03 adjuvant (G and H). SAS and AF03 adjuvants were found to induce equivalent responses to all HA-ferritin nanoparticles tested (Fig. 5A). The *x* axis indicates the panel of H1N1 influenza virus strains tested by reference year, from 1934 to 2013 (Table 1). Dashed lines mark the limit of detection (3.32). Horizontal gray bars mark the 1:40 to 1:80 ranges as a visual aid. Red asterisks indicate matched strains.

To study the immunogenicity of HA-Nps from specific viral strains, mice were immunized twice with the vaccine from the strain of interest, and sera were assayed 3 weeks after the second immunization using a hemagglutinin inhibition (HAI) assay. We used a panel of 16 representative influenza virus strains spanning 78 years of viral evolution (Fig. 3 and Table 1). High HAI titers were observed in all cases for the matched strain (CA09, NC99, FM47, and HK77) (Fig. 3A to D). Serological responses were also confirmed by neutralization of HA/NA pseudotyped lentiviruses (Table 2). The CA09 HA-Np elicited strong immune responses, but these were limited to contemporary strains after 2009 and a 1976 isolate that is also originated from swine and has close homology to CA09 (Fig. 3A) (23). NC99 HA-Np elicited potent HAI against influenza viruses from the late 1990s and early 2000s (Fig. 3B). The immunological response to FM47 HA-Np extended primarily to the matched strain, but it also showed modest cross-reactivity to the 1977 strain (Fig. 3C). This level of cross-reactivity has clinical relevance to the 1977 outbreak, which affected primarily people under 26 years of age, suggesting that exposure to influenza viruses from 1940 to 1950 outbreaks conferred protection against the 1977 strains (22). Interestingly, among the HA sequences, the HK77 HA-Np stood out because its cross-reactivity extended to strains from 1947, 1978, and 1983 (Fig. 3D). On the other hand, the immunological responses to MAL54 HA-Np and DV57 HA-Np were restricted to the matched strain (Fig. 3E and F and Table 2).

**TABLE 1:** Panel of H1N1 influenza virus strains used for HAI and MN assays, as indicated by reference year

H1N1 influenza panel
A/Puerto Rico/1934
A/Weiss/1/1943
A/FM/1/1947
A/Denver/1/1957
A/New Jersey/8/1976
A/USSR/90/1977
A/Brazil/11/1978
A/Chile/1/1983
A/Taiwan/1/1986
A/Texas/36/1991
A/Beijing/262/1995
A/New Caledonia/20/1999
A/Solomon Islands/6/2006
A/Brisbane/59/2007
A/California/07/2009
A/Bangladesh/2021/2012
A/Vietnam/3050/2013
A/Michigan/45/2015

**TABLE 2:**
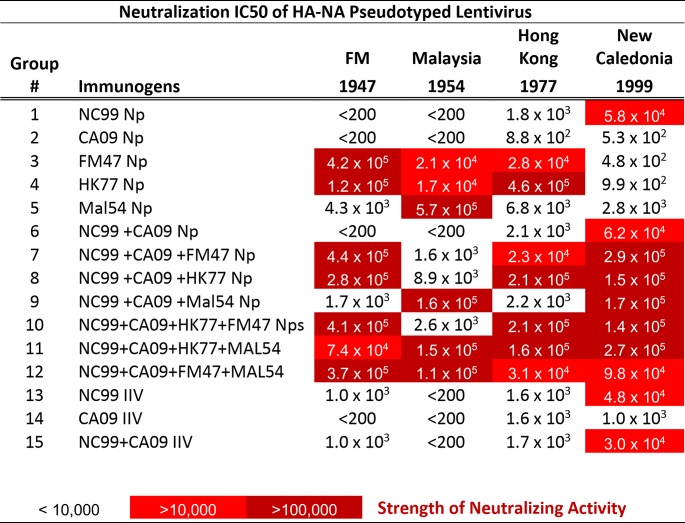
Neutralization activity toward HA/NA PsV in mice after two immunizations with HA-Nps administered as single components or in combinations^*a*^

a Mice (*n* = 5/group) were immunized twice with select immunogens, as indicated, with a 3-week interval. Five weeks later, serial dilutions of the serum from these mice were assayed for neutralization activity toward lentiviruses pseudotyped with HA and neuraminidase (NA) genes from the strains indicated by each column title. The IC_50_ values were calculated with GraphPad Prism software from these neutralization curves to determine the serum dilution factor that attains 50% neutralization of PsV. Strong neutralization activity was observed for the matched strains in all cases tested, and these values were used as thresholds for color coding. The combination of HA-Nps with complementary neutralization activities led to expanded cross-reactivity in an additive manner.

The cross-reactivity observed with COBRA P1 and COBRA X6 nanoparticles was consistent with their virus-like particle (VLP) counterparts (21). The immune response elicited by COBRA P1 HA-Np was similar to that of CA09 HA-Np (Fig. 3A and G), and COBRA X6 HA-Np showed an immune profile similar to that of NC99 HA-Np (Fig. 3B and H).

### Monovalent, bivalent, trivalent, and quadrivalent formulation of HA-ferritin nanoparticle vaccines.

**FIG 4:**
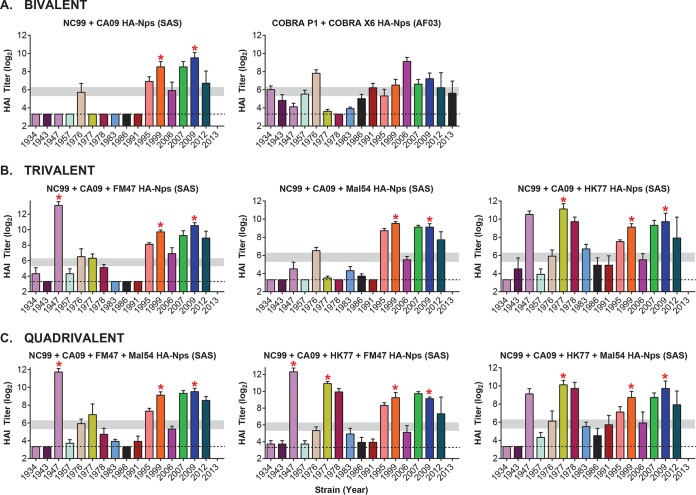
Immunogenicity of HA-ferritin nanoparticle vaccines administered to mice in bivalent (A), trivalent (B), or quadrivalent (C) combinations. HAI titers (log_2_) for a panel of divergent H1N1 influenza viruses are shown. Mice (*n* = 5) were immunized with the indicated HA-nanoparticle combinations at weeks 0 and 3 with adjuvants as indicated. The bivalent combination COBRA X6+COBRA P1 HA-Nps was tested with AF03 adjuvant, similar to the monovalent COBRA HA-Np evaluation described in the legend to Fig. 3. Likewise, the combinations of wild-type HA-ferritin nanoparticles used SAS adjuvant for consistency with the evaluation of the monovalent wild-type HA-Nps. Dashed lines mark the limit of detection (3.32). Horizontal gray bars mark the 1:40 to 1:80 ranges as a visual aid. Red asterisks indicate matched strains.

**FIG 5:**
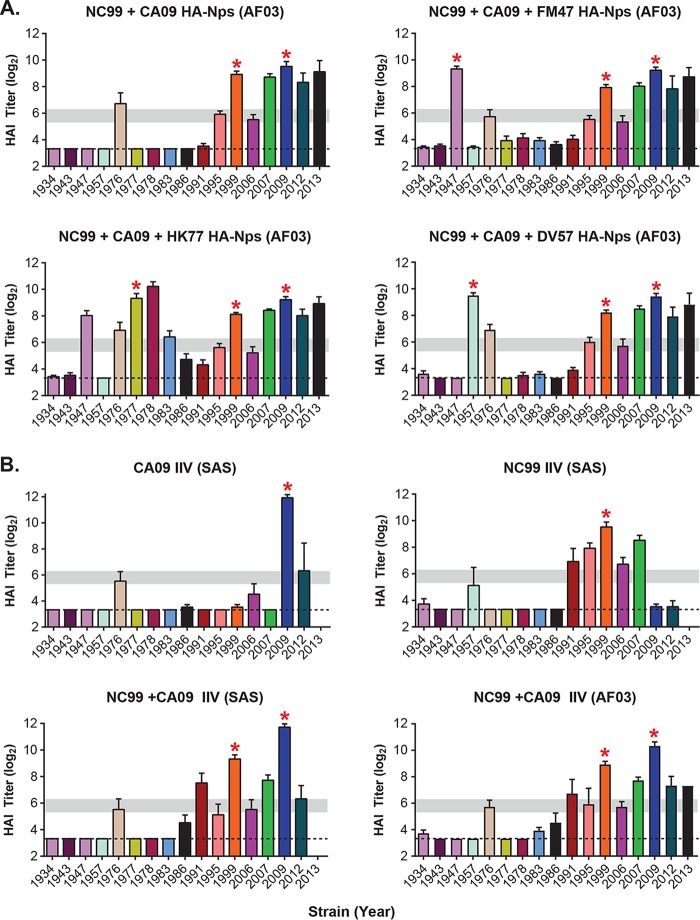
Immune breadth is largely determined by HA antigen selection. (A) Selected groups of HA-Nps were tested as bivalent or trivalent vaccines in mice by immunization at week 0 and week 3 with AF03 adjuvant. HAI titers (log_2_) were measured from mouse serum at week 6. (B) CA09 and NC99 influenza inactivated vaccines (IIV) were tested as monovalent and bivalent vaccines with either SAS or AF03 adjuvant, as indicated. Dashed lines mark the limit of detection (3.32). Horizontal gray bars mark the 1:40 to 1:80 ranges as a visual aid. Red asterisks indicate matched strains.

We evaluated the HAI cross-reactivity elicited by combinations of select HA-Nps. Mice were immunized and tested as described in Materials and Methods with bivalent, trivalent, or quadrivalent formulations. The bivalent combination of NC99 and CA09 HA-Nps showed expanded cross-reactivity relative to either monovalent vaccine (Fig. 4A). However, this bivalent combination did not elicit detectable antibody titers against the older divergent strains from 1934 to 1957 and 1977 to 1991. The immunogenicity of the COBRA X6 and COBRA P1 bivalent combination followed the same trend (Fig. 4A). This combination showed increased breadth compared to that of the NC99/CA09 bivalent vaccine, although HAI titers against several strains were moderate (Fig. 4A). For the trivalent combinations, inclusion of a third component to NC99 and CA09 HA-Nps increased cross-reactivity when the third component was either FM47 HA-Np or HK77 HA-Np, but MAL54 HA-Np did not enhance breadth (Fig. 4B). Addition of a fourth component in the quadrivalent formulations resulted in no additional cross-reactivity breadth compared to that of the optimal trivalent combination of NC99, CA09, and HK77 HA-Nps (Fig. 4C). Comparable results were observed when AF03 adjuvant was used instead of the Sigma adjuvant system (SAS) (Fig. 5A), and similar HAI profiles were obtained with NC99 and CA09 immunogens delivered as nanoparticles (Fig. 3A and B, 4A, and 5A) or egg-produced inactivated influenza vaccines using a normalized dose of HA (Fig. 5B). Importantly, we did not find evidence for antigenic competition or enhancement by coadministration of different HA-Nps. Our data suggest that cross-reactivity profiles are additive for cases in which there is a high degree of complementarity in their individual HAI profiles.

**FIG 6:**
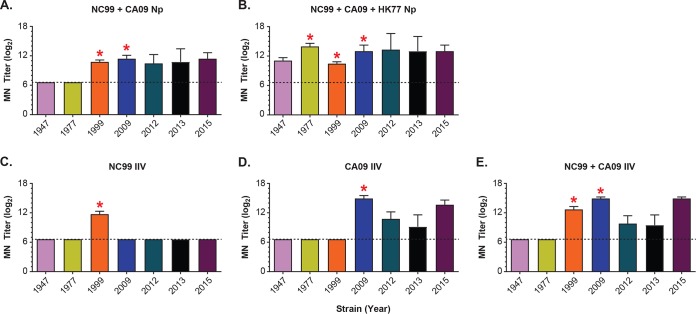
Microneutralization of homologous and heterologous H1N1 influenza virus strains by serum from mice at week 5 postimmunization. (A to E) MN titer (log_2_) for a panel of divergent H1N1 influenza viruses by serum from mice (*n* = 5) immunized with the indicated HA-nanoparticle combinations or egg-produced IIV vaccine monovalent or bivalent combinations, at weeks 0 and 3, with SAS adjuvant. Red asterisks indicated matched strains. The limit of detection is indicated with dashed lines at 1:100 (log_2_ scale of 6.6) based on the lowest serum dilution tested. Red asterisks indicate matched strains.

We confirmed the HAI assay observations by testing *in vitro* microneutralization (MN) activity of mouse serum from selected immunization groups against a panel of 7 H1N1 influenza viruses taken from those listed in Table 1 (Fig. 6A to E). We found that the bivalent vaccine NC99+CA09 HA-Nps neutralized matched or closely related influenza viruses from 1999, 2009, 2012, 2013, and 2015 (Fig. 6A). Consistent with the HAI results, this bivalent combination did not induce detectable neutralization activity (<1:100) against older strains, such as 1947 and 1977 influenza viruses. A similar trend was observed with monovalent NC99 or CA09 or bivalent NC99+CA09 IIV (Fig. 6C to E). Addition of the HK77 HA-Np extended breadth of the bivalent vaccine to neutralize both the 1947 and 1977 viruses (Fig. 6B). These results confirm that extended immune breadth can be achieved against divergent H1N1 influenza viruses using select combinations of antigens with recombinant HA-ferritin nanoparticles.

### Protection of trivalent vaccines against challenge in ferrets.

**FIG 7:**
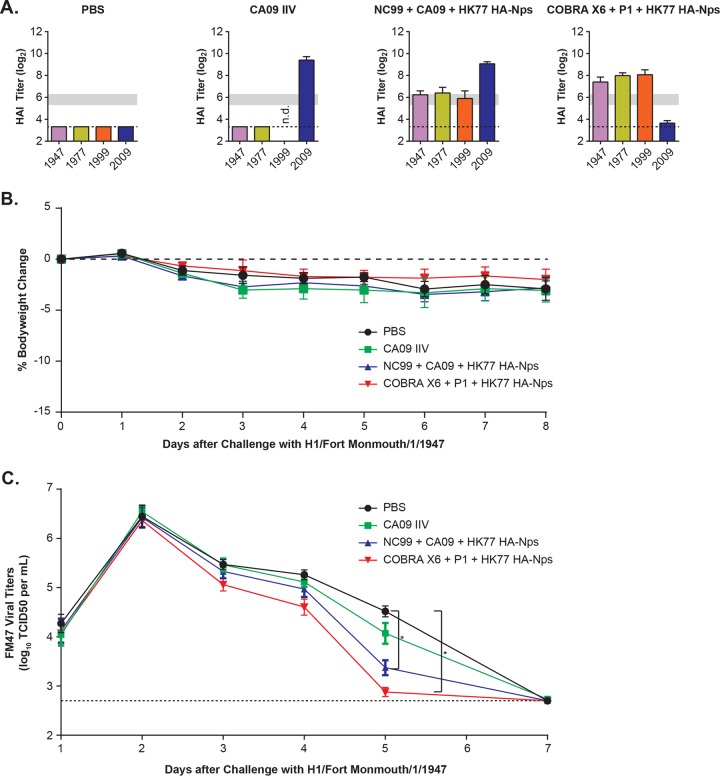
Heterologous influenza virus challenge study following immunization using HA-ferritin nanoparticle cocktails in ferrets. (A) HAI titers (log_2_) of serum from ferrets (*n* = 12 per group) following two immunizations with immunogens and AF03 adjuvant: group 1, PBS alone; group 2, A/California/7/2009 IIV; group 3, NC99+CA09+HK77 HA-Nps; group 4, COBRA X6+COBRA P1+HK77 HA-Nps. The dashed line indicates the limit of detection. Horizontal gray bars mark the 1:40 to 1:80 ranges as a visual aid. (B) Heterologous challenge of ferrets immunized with HA-ferritin nanoparticle cocktails or PBS alone using 1947 Fort Monmouth virus. The challenge was performed 4 weeks after the last immunization by intranasal inoculation with 1 ml of A/Fort Monmouth/1/1947 virus, with a TCID_50_ of 10^4.65^. Ferrets did not show significant weight loss after challenge. The dashed line indicates 0% change in weight. (C) Virus titers were quantified in the nasal washes over a time course after challenge. The dashed line indicates the assay limit of detection. Viral titers were significantly reduced at day 5 postchallenge in ferrets immunized with HA-Np combinations, but not with CA09 IIV, compared to vehicle (PBS) control by one-tailed unpaired *t* test and by one-way ANOVA [*F*(3, 44) = 5.18, *P* = 0.00375]. *, *P* ≤ 0.001 by Student's *t* test. The dashed line indicates the limit of detection.

The efficacy of the HA-Np combinations with optimal breadth was tested in ferrets, an animal model relevant to human disease. We immunized ferrets (*n* = 12 per group) with either phosphate-buffered saline, CA09 inactivated influenza vaccine (IIV), a trivalent wild-type HA-Np combination (NC99+CA09+HK77), or a combination of COBRA P1+COBRA X6+HK77 HA-Nps. After two immunizations, significant HAI titers against the matched strains were confirmed (Fig. 7A). Both groups of ferrets immunized with nanoparticle combinations showed significant HAI titers against FM47, HK77, and NC99. CA09 IIV did not elicit cross-neutralizing titers against FM47 and HK77 strains in ferrets, as observed in mice. After immunization, the ferrets were challenged with an unmatched divergent strain, H1/FortMonmouth/1/1947 virus, animals were monitored for clinical signs, and viral titers were quantified from nasal washes following the challenge. The FM47 strain did not induce significant weight loss in any group, including the unvaccinated control group (Fig. 7B), consistent with observations reported by others (24). However, FM47 successfully replicated in all groups, and it was cleared 1 week after infection (Fig. 7C). The ferret cohorts that received either trivalent NC99+CA09+HK77 or COBRA-P1+X6+HK77 nanoparticle combinations cleared the virus faster than the control group, displaying significantly reduced viral titers at day 5 postinfection (*P* ≤ 0.001) (Fig. 7C). In contrast, the CA09 IIV-immunized group did not clear virus faster than the phosphate-buffered saline (PBS)-immunized control group. These data suggest that the trivalent combinations stimulate effective HAI responses that also protect against divergent viral challenge in an animal model of infection relevant to human disease.

## DISCUSSION

Recombinant HA vaccines have been shown to be safe and effective in protecting against influenza virus infection, similar to traditional influenza vaccines (5, 10). The use of recombinant proteins as immunogens provides versatility in the presentation of alternative antigens, because it does not require the rescue of live viruses. The ferritin nanoparticle platform has been used to display diverse viral antigens, such as influenza HA (11), EBV gp220 (25), and HIV envelope (26). In all of these cases, multivalent presentation on ferritin nanoparticles outperformed the soluble protein immunogens. HA-ferritin nanoparticles are currently being evaluated in a human clinical trial (ClinicalTrials registration no. NCT03186781) as an alternative to egg- or cell-based split or inactivated influenza vaccines.

This study underscores the potential of the HA-ferritin nanoparticle vaccine platform. In addition to their ability to effectively present highly conserved antigens, they can be readily produced in large quantities, with homogeneity, and with diverse influenza virus HAs using standard molecular cloning strategies. All monovalent HA-Nps tested elicited potent humoral responses that neutralized the cognate strains. However, their cross-reactivity appears to be an inherent property of HA antigens that could not be predicted *a priori* based on sequence information. We found that nanoparticles displaying HAs from NC99, CA09, and HK77, as well as the two COBRA antigens, elicited high HAI responses against multiple mismatched strains, whereas other immunogens tested, such as FM47, MAL54, and DV57 Nps, induced primarily strain-specific responses.

For this analysis we relied on HAI assays, because this functional assay has an established correlate of protection in humans and is also used to validate new influenza vaccines (27). We corroborated the HAI observations by testing microneutralization activity of mouse serum from selected immunization groups against a panel of 7 divergent strains. We observed that the cross-reactivity profiles of divergent HA immunogens were mutually complementary, and that the combination of these HA-Nps led to an additive effect on the immune breadth profile. Importantly, there was no evidence of interference, or antigenic competition, when mixing up to 4 components. Finally, we found that trivalent combinations of select HA-Np immunogens induced faster viral clearance than the H1N1 standard of care, CA09 IIV vaccine, following challenge with a divergent virus.

Taken together, our observations suggest that a vaccine providing broad coverage against H1 influenza is feasible based on a combination of select immunogens. The combination of NC99 and CA09 immunogens increased the breadth of H1N1 neutralization dating back to the mid-1990s, and the addition of the HK77 immunogen as a third component further extended coverage to most of the seasonal viruses that circulated over the past 80 years. Finally, we found that the nonnatural COBRA HAs, X6 and P1, extended coverage to most strains tested when used in combination. The lack of detectable antibody titers to 1947, 1977, 1978, and 1983 strains by the COBRA P1+COBRA X6 combination suggests that it could be further improved by addition of the HK77 immunogen or by specifically including sequences from this time period to generate a new COBRA candidate. Beyond antigen selection, further studies are necessary to elucidate the optimal dosing and vaccination regimen that will lead to broadly protective immune responses in humans.

The advent of high-throughput DNA sequencing and bioinformatics over the past decade has led to vast databases of viral sequences that have affected human populations across the world and over the past century (28). This information could be exploited to fight viruses such as influenza that use genetic divergence as a strategy to evade immune protection (28, 29). It has been postulated that influenza viruses use nonconserved epitopes as decoys to divert the immune system from sequences that are structurally or functionally restricted from divergence (30). For example, the COBRA approach attempted to decrypt the HA conserved epitopes through the sequence averaging of multiple strains (21, 29, 31). However, the cross-reactivity of the immune responses elicited by evolutionarily divergent wild-type HA sequences has not previously been characterized systematically. By expanding the panel of HA immunogens and HAI assays and using combinations of specific influenza virus strains, it is possible to improve the functional cross-reactivity of the vaccine response. This understanding will facilitate the development of next-generation H1 vaccines, as it advances the goal of universal influenza protection.

## MATERIALS AND METHODS

### Vector construction.

All sequences were codon optimized for expression in human cell lines. The HA ectodomain from various H1N1 strains was genetically fused to the N terminus of Helicobacter pylori-bullfrog hybrid ferritin, described in reference 25. HA sequences from the following influenza virus strains were used to construct the HA-nanoparticles: A/Fort Monmouth/1-JY2/1947 (GenBank accession no. CY147342, amino acids 1 to 518, Y108F), A/Malaysia/302/1954 (accession no. CY009340.1, amino acids 1 to 518, Y108F), A/Denver/1/1957 (accession no. CY008988, amino acids 1 to 517, Y108F), A/Hong Kong/117/1977 (accession no. CY009292, amino acids 1 to 518, Y108F), A/New Caledonia/20/99 (accession no. AHJ09883.1, amino acids 1 to 518), A/California/4/2009 (accession no. AHJ09884.1, amino acids 1 to 518), COBRA P1 (Seq Id 2; amino acids 1 to 518, Y108F) (32), and COBRA X6 (amino acids 1 to 517, Y108F) (33). The HA-ferritin genes were cloned into the XbaI/BamHI sites of SIB002 vector for mammalian expression, with a GCCACC Kozak sequence in front of the ATG start codon.

### Sequence alignments.

Align X tool of Vector NTI software was used to align HA sequences. A guide tree or dendrogram was generated with Align X, which uses the neighbor-joining method of Saitou and Nei to calculate a matrix of distances between all pairs of sequences in the analysis. The calculated distances are related to the divergence between the sequences.

### Protein expression and purification.

HA-ferritin plasmids were purified with the PowerPrep kit (NP100009; Origene) and used to transfect Expi293 cells (A14635; ThermoFischer). We used the FectoPRO DNA transfection reagent (116-100; Polyplus) using standard conditions (0.5 μg of DNA/ml, 0.75 μl FectoPRO reagent/ml, and 0.45 μl of enhancer/ml). Nanoparticles were harvested from Expi293 supernatant by centrifugation, at 3,488 × *g* for 15 min at 4°C, 4 days after transfection and filtered through a 0.45-μm vacuum-driven filter unit (167-0045; Thermo Scientific). HA-ferritin nanoparticles were passed through a Q-Sepharose fast flow column (17051001; GE) by gravity flow. The flowthrough was collected and diluted 3× with water, and pH was adjusted by adding Tris buffer, pH 8.5, at a final concentration of 50 mM. The samples were then loaded onto another Q-Sepharose column (HiTrap Q HP; 17115401; GE), and proteins were eluted over a NaCl gradient with 0 to 60% mixing of buffer A (50 mM Tris, pH 8.5, 5 mM NaCl) and buffer B (50 mM Tris, pH 8.5, 1 M NaCl) over 30 column volumes. The HA-nanoparticle protein fractions were collected and concentrated with an Amicon Ultra-15 centrifugal filter unit (UFC910024; Millipore) and further purified by size exclusion chromatography with a Superose 6 column, PG XK 16/70, 60 to 65 cm (no. 90100042; GE), in phosphate-buffered saline. The final fractions were concentrated and filter sterilized through a 0.22-μm filter (SLGV004SL; Millipore). We used endotoxin-free solutions and tested the final proteins using the Charles Rivers Endosafe PTS instrument with LAL cartridges with a limit of detection of 0.05 EU/ml.

### Electron microscopy (EM) and DLS.

HA-ferritin nanoparticle samples were adsorbed for 1 min to a carbon-coated grid that had been made hydrophilic by a 30-s exposure to a glow discharge. Excess liquid was removed with filter paper (Whatman number 1), and the samples were stained with 0.75% uranyl formate for 30 s. After removing the excess uranyl formate with a filter paper, the grids were examined in a TecnaiG^2^ Spirit BioTWIN, and images were recorded with an AMT 2k charge-coupled device camera. Dynamic light scattering (DLS) was measured on Wyatt's DynaPro plate reader II at 25°C.

### Mouse immunizations.

Animal experiments were carried out in accordance with all federal regulations in an AAALAC-accredited facility per the standards of the *Guide for the Care and Use of Laboratory Animals* (34). Protocols were reviewed and approved for scientific rigor and animal welfare by the Institutional Animal Care and Use Committee. Two days prior to the first immunization, serum was collected from each mouse, and serum pools of 5 mice were tested for influenza preexposure by enzyme-linked immunosorbent assays (ELISA). All mice used in this study were naive to H1N1 influenza virus, as defined by undetectable ELISA signal (<0.1 absorbance) at 1:40 serum dilution on ELISA plates coated with A/California/4/2009 HA trimers. BALB/c mice (5/group) were immunized at weeks 0 and 3 with 220 ng of HA-ferritin nanoparticles (170 ng of HA content) or with an HA-matched dose of egg-produced inactivated influenza vaccine (IIV), mixed 1:1 with adjuvant immediately before intramuscular injection (50 μl per hind leg). The Sigma adjuvant system (SAS; also known as Ribi; no. S6322-1vl) or AF03 (Sanofi Pasteur) was used as indicated. For bivalent, trivalent, and quadrivalent combinations, 220 ng of each nanoparticle was premixed before injection. Sera were collected at 2 and 3 weeks after the second immunization.

### Ferret immunizations.

Forty-eight (24 male and 24 female) domestic ferrets (*Mustela putorious furo*) were purchased from Triple F Farms (Sayre, PA) and housed and cared for by Bioqual, Inc., in compliance with USDA regulations and the Animal Welfare Act. The ferrets were approximately 16 to 18 weeks of age at time of delivery (approximately 26 to 28 weeks at time of challenge). All 48 ferrets passed an influenza prescreening HAI test [HAI undetectable at 1:10 for B/Brisbane/60/08, B/Phuket/3073/2013, A/California/07/2009 (H1N1), and A/Hong Kong/4801/2014 (H3N2)]. Groups of 12 ferrets were immunized at weeks 0 and 4 with either phosphate-buffered saline, 3.35 μg of HA-ferritin nanoparticles as indicated, or a matched dose of A/California/7/2009 inactivated influenza vaccine. Before intramuscular injection, these immunogens were mixed 1:1 with AF03 adjuvant for a 1-ml final injection volume. The influenza virus challenge was performed 4 weeks after the second immunization by intranasal inoculation with 1 ml of A/Fort Monmouth/1/1947 virus with a 50% tissue culture infectious dose (TCID_50_) of 10^4.65^. Clinical signs were monitored daily for 2 weeks, and nasal washes were collected daily for 7 days postchallenge and tested for viral load by standard TCID_50_ assay.

### HAI assay.

Influenza virus seed stocks used in HAI assays were from the Centers for Disease Control and Prevention (Atlanta, Georgia, USA). The HAI assay was performed by following standard methodology established by the WHO, which tests the quantity of serum required to interfere with the agglutinating activity of 4 agglutinating units of virus. Briefly, the immune sera were pretreated with receptor-destroying enzyme by diluting one part serum with three parts enzyme and incubated overnight in a 37°C water bath. The enzyme was inactivated by a 30-min incubation at 56°C, followed by addition of six parts PBS to a final dilution of 1/10. HAI assays were performed in V-bottomed 96-well microtiter plates with 4 HA units of virus (HAU) in 0.5% turkey red blood cells. The HAI titer was determined as the highest dilution of serum resulting in complete inhibition of hemagglutination. Preimmune sera were used as a negative control for each influenza strain tested in HAI assays.

### MN assay.

MN assay was performed by Bioqual, Inc., by following Standard Operating Procedure number BV-012 and the 2013 WHO Laboratory Procedures guidelines. Briefly, serum samples collected from mice 5 weeks after the first immunization, with a boost at 3 weeks, were treated with receptor-destroying enzyme II for 18 to 20 h and heat inactivated at 56°C for 40 min prior to MN testing. Serial dilutions of serum were incubated with influenza virus, as indicated by reference year (strains are listed in Table 1), for 1 h at 37°C before being transferred to 96-well plates with MDCK target cells. After 2 days of incubation, influenza virus was detected by ELISA to calculate the virus neutralization endpoint antibody titer. Reference serum, virus back-titration, and preimmune serum pools for each group were included as controls.

### PsV neutralization assay.

The pseudotyped virus (PsV) neutralization assay was performed as previously described (11). Briefly, lentiviruses were packaged in HEK293T cells by transfection of 5 plasmids, 400 ng of HA, 100 ng of NA, 50 ng of TMPRSS2, 7 μg of CMVΔR8.2, and 7 μg of pHR′CMV-Luc with the Profection mammalian transfection kit (E1200; Promega). Viral particles were collected 48 and 72 h posttransfection and filtered through a 0.45-μm filter. Antisera at the indicated dilution were preincubated with a fixed amount of lentivirus and used to infect target 293A cells. Infection was quantified 72 h later with the Promega luciferase assay system (no. E1500). Fifty percent inhibitory concentrations were calculated with GraphPad Prism from neutralization curves. Preimmune sera were used as a negative control for each influenza strain tested in PsV assays.

### Statistical analysis.

Error bars represent the standard error of the mean obtained from assaying samples from each animal in a given treatment group (*n* = 5 for mice and *n* = 12 for ferrets). Student's *t* test was calculated with Microsoft Excel. Analysis of variance (ANOVA) was calculated with VassarStats (http://vassarstats.net/anova1u.html).
